# Volatile-Mediated Signalling Between Potato Plants in Response to Insect Herbivory is not Contingent on Soil Nutrients

**DOI:** 10.1007/s10886-023-01445-y

**Published:** 2023-07-18

**Authors:** Lucía Martín-Cacheda, Carla Vázquez-González, Sergio Rasmann, Gregory Röder, Luis Abdala-Roberts, Xoaquín Moreira

**Affiliations:** 1 Misión Biológica de Galicia (MBG-CSIC), Apartado de correos 28, Pontevedra, Galicia, 36080 Spain; 2 Department of Ecology and Evolutionary Biology, University of California-Irvine, Irvine, CA 92697 USA; 3 Institute of Biology, University of Neuchâtel, Rue Emile-Argand 11, Neuchâtel, 2000 Switzerland; 4 Departamento de Ecología Tropical, Campus de Ciencias Biológicas y Agropecuarias, Universidad Autónoma de Yucatán, Apartado Postal 4-116, Itzimná, Mérida, Yucatán 97000 México

**Keywords:** Fertilization, Plant-plant communication, Plant-herbivore interaction, *Solanum tuberosum*, *Spodoptera exigua*, Volatile organic compounds

## Abstract

Plant-plant signalling via volatile organic compounds (VOCs) has been studied intensively, but its contingency on abiotic conditions (e.g., soil nutrients, drought, warming) is poorly understood. To address this gap, we carried out a greenhouse experiment testing whether soil nutrients influenced signalling between potato (*Solanum tuberosum*) plants in response to insect leaf herbivory by the generalist caterpillar *Spodoptera exigua*. We placed pairs of plants in plastic cages, where one plant acted as a VOC emitter and the other as a receiver. We factorially manipulated soil nutrients for both emitter and receiver plants, namely: unfertilized (baseline soil nutrients) vs. fertilized (augmented nutrients). Then, to test for signalling effects, half of the emitters within each fertilization level were damaged by *S. exigua* larvae and the other half remained undamaged. Three days after placing larvae, we collected VOCs from emitter plants to test for herbivory and fertilization effects on VOC emissions and placed *S. exigua* larvae on receivers to test for signalling effects on leaf consumption and larval mass gain as proxies of induced resistance. We found that herbivory increased total VOC emissions and altered VOC composition by emitter plants, but these effects were not contingent on fertilization. In addition, bioassay results showed that receivers exposed to VOCs from herbivore-damaged emitters had lower levels of herbivory compared to receivers exposed to undamaged emitters. However, and consistent with VOC results, fertilization did not influence herbivore-induced signalling effects on receiver resistance to herbivory. In sum, we found evidence of *S. exigua*-induced signalling effects on resistance to herbivory in potato plants but such effects were not affected by increased soil nutrients. These results call for further work testing signalling effects under broader range of nutrient concentration levels (including nutrient limitation), teasing apart the effects of specific nutrients, and incorporating other abiotic factors likely to interact or covary with soil nutrients.

**Supplementary Information:**

The online version contains supplementary material available at 10.1007/s10886-023-01445-y.

## Introduction

Plants emit complex blends of volatile organic compounds (VOCs) that are perceived by neighbouring plants (“receivers” hereafter), a phenomenon commonly termed “plant-plant communication” (Heil and Karban 2010; Karban et al. 2014). In this form of plant signalling, exposure to VOCs from herbivore-damaged plants (“emitter” hereafter) prime defences and increase resistance in undamaged neighbouring plants (Heil and Karban 2010; Vlot et al. 2020). Plant communication in response to herbivory is thought to be highly specific and context-dependent, as plants modify their VOC emissions according to the type of stress they are facing (Moreira and Abdala-Roberts 2019), including changes in the abiotic environment (e.g., soil nutrients, water availability, temperature, etc.) (Chen et al. 2008; Giron-Calva et al. 2017; Scott et al. 2019; Quijano-Medina et al. 2021). Accordingly, recent work has found evidence for abiotic context-dependency in herbivore-induced VOCs affecting plant-plant signalling, mainly in response to changes in water availability (Pezzola et al. 2017; Catola et al. 2018; Vázquez-González et al. 2022). However, the influence of abiotic forcing on herbivore-driven plant signalling still remains poorly studied (Moreira and Abdala-Roberts 2019; Karban 2021), and several abiotic factors have virtually not been tested within this context.

The effects of soil nutrient availability have received much attention in plant defence research (Coley et al. 1985; Herms and Mattson 1992; Hahn and Maron 2016). Trade-offs between plant defences (e.g., secondary metabolites production) and other functions (e.g., growth, reproduction) have been invoked as a probable mechanism driving changes in allocation to plant defence under varying levels of soil resource availabily. For example, the Growth-Differentiation Balance Hypothesis (GDBH, Loomis 1932) predicts a physiological trade-off between the production of secondary metabolites and the demand for photosynthates during plant development (Herms and Mattson 1992), such that plants growing under high resource availability will prioritize growth over defences (Chapin III 1980; Bazzaz et al. 1987). However, research has also shown that plants in many instances can allocate simultaneously to growth and defence under high resource availability (Hahn and Maron 2016), and there may even be positive correlations between growth and defence (Hahn and Maron 2016; López-Goldar et al. 2020). To date, most research testing mechanisms of soil nutrient effects on plant defences has involved non-volatile secondary metabolites and physical defences (reviewed by Koricheva 2002; Hahn and Maron 2016), whereas work on VOCs is much less common (but see Gouinguené and Turlings 2002; Schmelz et al. 2003; Ibrahim et al. 2008). This important gap remains to be addressed, in particular with respect to nutrient effects on induced VOCs in response to herbivory, the underlying mechanisms for such effects (e.g., growth-defence associations), and the extended consequences for plant-plant signalling.

In this study, we tested for the effects of soil nutrients on herbivore-induced VOC signalling between potato (*Solanum tuberosum*) plants. For this, we carried out a greenhouse experiment in which we placed pairs of plants (i.e., emitter and receiver) in plastic cages and factorially manipulated soil nutrient levels for both emitter and receiver plants by applying a fertilization treatment. We then assigned half of the emitters within each level of emitter fertilization to damage by larvae of the generalist herbivore *Spodoptera exigua* and the other half were left undamaged. We measured total emission and composition of VOCs released by emitter plants to test for herbivory (i.e., induction) and fertilization effects and their interaction on VOC emissions. We then conducted a bioassay of herbivory (i.e., percentage of leaf area removed) and larval performance (growth) on receiver plants to test for VOC-mediated signalling effects on receiver resistance and its contingency on soil nutrients. One prediction is that increased nutrient levels under fertilization will weaken signalling effects due to growth-defence trade-offs whereby plants prioritize growth over defences by reducing emitter VOC induction and/or by weakening receiver induced responses. Alternatively, fertilization could strengthen signalling by dampening any such trade-off or promote a positive correlation between functions by enhancing emitter VOC induction and/or receiver induced defences. Overall, by addressing the effects of soil nutrients on VOC-mediated plant-plant signalling, this study fills a key gap in knowledge pertaining the abiotic context-dependency of plant signalling and its underlying mechanisms. Findings can also inform pest and soil management strategies in potato agroecosystems.

## Materials and Methods

### Study System

*Solanum tuberosum* L. (Solanaceae) is an herbaceous plant that grows up to 60 cm tall and propagates by seeds and tubers. Its domestication can be traced back to c. 8000 years ago in the central Andes (Peru-Bolivia) (Hijmans and Spooner 2001), and it was introduced in Europe in the second half of the 16th century. At present, it is one of the most important crops in terms of human consumption, with more than 4000 edible varieties and an annual production of more than 359 million tonnes (FAOSTAT 2020).

The intensification of agricultural practices has resulted in marked increases in damage by pests and pathogens associated with potato. One of the most economically important pests on this crop is the beet armyworm (*Spodoptera exigua* (Hubner), Lepidoptera: Noctuidae), a generalist insect that feeds on leaves and tubers causing significant reductions in plant growth and yield (Brown and Dewhurst 1975). Importantly, previous work by our group has shown that leaf damage by *S. exigua* significantly increases total VOC emission as well as leads to compositional changes in VOC blends in young potato plants (Vázquez-González et al. 2022; Martín-Cacheda et al. 2023). In turn, these herbivore-induced changes in VOC emissions trigger plant-plant signalling which results in heightened resistance to herbivory in neighbouring undamaged plants (Vázquez-González et al. 2022; Martín-Cacheda et al. 2023).

### Experimental Design

In November 2021, we sowed 160 tubers of *S. tuberosum* (Baraka cultivar) individually in 4-L pots containing potting soil with peat (Gramoflor GmbH & Co. KG Produktion, Vechta, Germany). This soil was chosen due to its similarity with the agricultural soil of north-western Spain (Carballas et al. 2016), and was rich in organic matter and had a basal nutrient level of: 50–300 mg/l N, 80–300 of mg/l P_2_O_3_ and 80–400 mg/l K_2_O. Plants were grown in a glasshouse under controlled light (minimum 10 h per day, Photosynthetically Active Radiation = 725 ± 19 µmol m^− 2^ s^− 1^) and temperature (10 °C night, 25 °C day), and watered three times a week. Once plants were three weeks old, we assigned half to one of two nutrient level treatments: control (unfertilized) or fertilized. We watered plants every three days with 500 ml of water or water plus 2.5 ml of liquid fertilizer (Fertimon Red 2(N, nitrogen)-10(P, phosphorus)-26 (K, potassium) + 2.8MgO + Micros), respectively during a three-week period. This treatment was aimed to mimic typical doses of NPK fertilization practices in potato crops, particularly for the Baraka cultivar used (García 2014), and resulted in an increase in nitrogen, phosphorus and potassium of 150, 750 and 1200 mg, respectively per plant. It is important to note that despite having nutrient reserves in the tuber, potato plants can become strongly nutrient-limited due to their shallow rooting system (Koch et al. 2020).

**Fig. 1:**
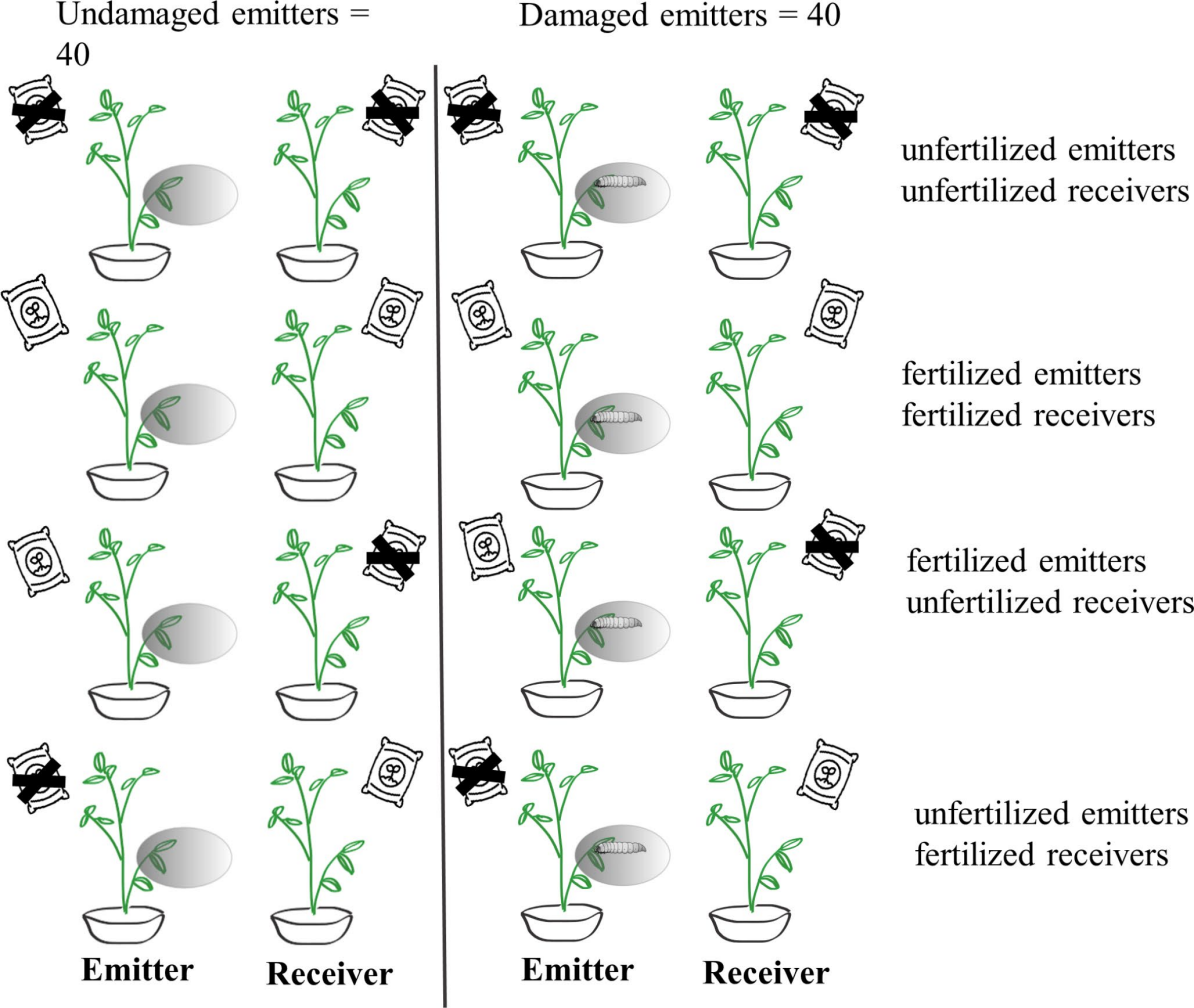
Experimental design to test for effects of soil nutrients on communication between potato (*Solanum tuberosum*) plants (N = 80). We paired potato plants designated as emitters and receivers, with half of the emitters receiving damage by *Spodoptera exigua* larvae (i.e., herbivore-damaged plants) and half serving as undamaged controls. Both emitter and receiver plants were also subject to two fertilization treatments (fertilized vs. unfertilized), resulting in a three-way factorial design

After applying the fertilization regime, we paired potato plants in 37.5 × 37.5 × 96.5 cm plastic cages to prevent VOC-mediated cross-communication between replicates. Each cages had two frontal holes covered with a mesh allowing airflow. One plant of each pair acted as the emitter and the other as the receiver. Within each cage, plants were placed 20 cm apart, avoiding direct physical contact. Adjacent cages were spaced by 2 m to prevent VOC cross-signalling among replicates. We then randomly assigned half of the emitter plants of each level of fertilization to one of following herbivore damage treatments: (1) subjected to *S. exigua* damage (i.e., induced plants) or (2) undamaged (control) plants (Fig. 1). Plants were six weeks old at the time this treatment was applied. Specifically, for herbivore-damaged plants we placed one third-instar larvae of *S. exigua* on each of two fully expanded leaves per plant using a fine paintbrush and covered these leaves with a nylon bag to prevent herbivore dispersal. For undamaged plants, we covered two fully expanded leaves with a nylon bag but did not add larvae. In total, the experiment consisted of 80 replicates (cages) allocated in the following way: 40 per emitter damage level, 40 per emitter or receiver fertilization level, 20 for each emitter by receiver fertilization combination, and 10 per emitter fertilization by receiver fertilization by emitter damage combination. In addition to VOC and leaf damage measurements (see ahead), we also recorded height and basal stem diameter for of all plants as proxies of plant growth and ran a general linear model in R software version 4.1.2 (R Core Team 2020) to test for the effects of fertilization (two levels: unfertilized vs. fertilized) on plant growth. Fertilized plants were 8.9% taller (unfertilized: 41.63 ± 0.55 cm; fertilized: 45.37 ± 0.6 cm; F_1,157_ = 18.63, *P* < 0.001) and had a 17.5% greater basal stem diameter (unfertilized: 8.11 ± 0.19 mm; fertilized: 9.53 ± 0.23 mm; F_1,157_ = 22.04, *P* < 0.001) compared to unfertilized plants (Fig. S1).

Overall, the above experiment encompassed eight treatment combinations (i.e., unfertilized emitters/ unfertilized receivers, unfertilized emitters/fertilized receivers, fertilized emitters/fertilized receivers, and fertilized emitters/ unfertilized receivers; equal sample sizes for each one; see Fig. 1), a three-way factorial design allowing to test for both emitter and receiver-based sources of variation in fertilization effects on signalling (see also Vázquez-González et al. 2022).

### VOC Collection by Emitter Plants

After 72 h of *S. exigua* feeding, we removed all emitter plants from cages and collected aboveground VOCs from all the emitter plants following (Rasmann et al. 2011). Briefly, we bagged plants with a 2 L Nalophan bag, and trapped VOCs on a charcoal filter (SKC sorbent tube filled with Anasorb CSC coconut-shell charcoal) for two hours using a Sidekick 224-52MTX pump (0.25 L min^− 1^ airflow of technical air N_2_O_2_). We eluted traps with 150 µL dichloromethane (CAS#75-09-2, Merck, Dietikon, Switzerland) to which we had previously added one internal standard (tetralin CAS#119-64-2, 200 ng in 10 µL dichloromethane). We then injected 1.5 µL of the extract for each sample into an Agilent 7890B gas chromatograph (GC) coupled with a 5977B mass selective detector fitted with a 30 m × 0.25 mm × 0.25 μm film thickness HP-5MS fused silica column (Agilent, Santa Clara, CA, USA). We operated the injection into the GC in pulsed splitless mode (250 ºC, injection pressure 15 psi) with helium as the carrier gas. The GC oven temperature program was: 3.5 min hold at 40 ºC, 5 ºC min^− 1^ ramp to 230 ºC, then a 3 min hold at 250 ºC post run (constant helium flow rate 0.9 mL min^− 1^). The transfer line was set at 280 ºC. In the MS detector (EI mode), a 33–350 (m/z) mass scan range was used with MS source and quadrupole set at 230 ºC and 150 ºC, respectively. We identified volatile terpenes using commercial pure standards and comparing their Kováts indices, calculated relative to the retention times of a series of n-alkanes (C_8_-C_20_, Sigma-Aldrich, Merck KGaA, Darmstadt, Germany) analysed under the same chromatographic conditions, with those reported in the literature. It is important to note that, although our Kováts indices matched well with those previously reported, VOCs should be considered as ‘putative’ until confirmation with standards. We quantified total emission of individual VOCs using normalized peak areas and expressed it as nanograms per hour (ng h^− 1^). We obtained the normalized peak area of each individual compound by dividing their integrated peak areas by the integrated peak area of the internal standard (Abdala-Roberts et al. 2022), in order to standardize for varations in the sample volume during the elution process. Reported values for individual VOCs should thus be considered as tetralin-equivalent nanograms of compound released by each plant per hour. The total emission of VOCs of each sample (i.e., emitter plant) was then obtained by summing the concentrations of individual VOCs. Due to an elution issue, we removed three VOC samples and therefore used 77 samples for statistical analyses (20 undamaged-unfertilized, 18 undamaged-fertilized, 19 damaged-unfertilized and 20 damaged-fertilized emitter plants).

### Bioassay of Induced Resistance in Receiver Plants

The same day after collecting emitter VOCs, we conducted a bioassay on all receiver plants to test whether exposure to VOCs from damaged emitters boosted resistance against herbivory and whether any such effect was contingent on fertilization. For this, we placed one third-instar *S. exigua* larvae on each of two fully expanded leaves per receiver plant following the same procedure described above for the emitter herbivore damage treatment. Prior to the bioassay, we weighed all larvae to the nearest 0.0001 g to control for larval initial mass. After 24 h of feeding, we collected leaves and photographed them with a Samsung Galaxy A30s (25 effective megapixels, 4× digital zoom). We estimated the percentage of leaf removed using the mobile application BioLeaf - Foliar Analysis™ (Brandoli Machado et al. 2016). We then weighed all larvae again to estimate larval mass gain (final mass – initial mass).

### Statistical Analyses

*Effects of herbivore damage and soil nutrients on emitter VOCs*. We ran general linear models to test the effects of emitter herbivore damage treatment (undamaged vs. herbivore-damaged), fertilization treatment (unfertilized vs. fertilized), and their interaction (all fixed effects) on total VOCs released by emitter plants, as well as on each individual compound. We also included plant height as a covariate to account for differences in plant size potentially affecting the amount of VOCs emitted. For tests of individual compounds, we performed *P*-value adjustments using the false discovery rate for *P* < 0.05 to avoid inflating Type I error due to multiple testing (Benjamini and Hochberg 1995). In all cases, we used a normal error (identity as link) and log-transformed total VOC emission to achieve normality of residuals.

In addition, we ran a Permutational Multivariate Analyses of Variance (PERMANOVA) with 10,000 permutations to test for effects of emitter damage, fertilization treatment, and their interaction on VOC composition using individual compound abundances, i.e., qualitative variation in VOC emissions. To visualize these results, we performed a Principal Coordinate Analysis (PCoA) based on Bray-Curtis pairwise dissimilarities and graphed the centroids of each herbivore damage and fertilization treatment, separately (Moreira et al. 2021). We also identified influential VOCs, i.e., those having the strongest association with the first two ordination axes (R^2^ > 0.85 and R^2^ > 0.55, for herbivore damage and fertilization treatments respectively), and displayed these relationships using biplot arrows with the length scaled to R^2^ values.

*Effects of herbivore damage and soil nutrients on receiver resistance*. We ran general linear mixed models testing the effects of emitter herbivore damage (two levels: undamaged vs. herbivore-damaged), emitter fertilization (two levels: unfertilized vs. fertilized), receiver fertilization (two levels: unfertilized vs. fertilized), and all two- and three- way interactions (all as fixed factors) on the percentage of leaf removed and larval mass gain on receiver plants. The two-way interactions between emitter herbivore damage treatment and emitter and receiver fertilization were of main interest since they tested whether fertilization affected VOC herbivore-induced signalling effects on receiver resistance. Again, we included emitter and receiver plant height as covariates in both models to account for size differences that could affect signalling effects on receivers induced resistance. We also included plant as a random factor to control for the non-independence of each pair of leaves (for percentage of leaf area removed) or larvae (for larval mass gain) analysed per receiver plant. We log-transformed mean percentage of leaf area removed and larval mass gain to achieve normality of residuals.

We ran all statistical analyses in R software version 4.1.2 (R Core Team 2020). We implemented linear models and linear mixed models using the *lm* and *lmer* functions, respectively, from the *lmerTest* package (Kuznetsova et al. 2017). Model least-square means and standard errors (back-transformed for log-transformed data) are reported as descriptive statistics using the *lsmeans* function from the *lsmeans* package (Lenth 2016). Finally, we implemented PERMANOVA and ordination methods using the *adonis* and *capscale* functions respectively, both in the *vegan* package (Oksanen et al. 2016).

## Results

### Effects of Herbivore Damage and Soil Nutrient Nutrients on Emitter VOCs

**Fig. 2:**
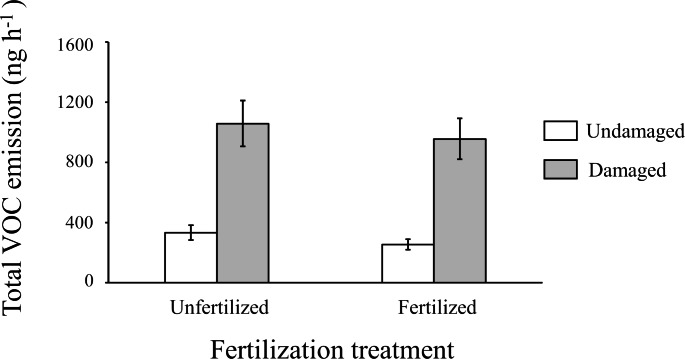
Effects of emitter herbivore damage treatment (undamaged vs. damaged by *Spodoptera exigua*) on the total emission of volatile organic compounds (VOCs, in nanograms per hour) produced by unfertilized vs. fertilized emitter potato (*Solanum tuberosum*) plants. Values shown are model back-transformed least-square means ± SE (N = 18–20)

We detected a total of 33 relevant VOCs in the headspace of potato plants (Table S1). We found a significant effect of emitter damage treatment on VOC emissions (Table 1), whereby herbivore-damaged plants produced 2.5 times more total VOCs than undamaged plants (mean ± SE: undamaged = 290.13 ± 29.21 ng h^− 1^; herbivore-damaged = 1005.77 ± 99.49 ng h^− 1^) (Fig. 2). On the other hand, soil fertilization and the emitter herbivore damage by fertilization interaction did not significantly affect VOC emissions (Table 1; Fig. 2). Similarly, analyses of individual compounds showed that emitter plants damaged by *S. exigua* consistently increased the emission of all VOCs emitted (Table S1), whereas fertilization and the interaction were consistently non-significant (Table S1).

**Table 1:** Effects of emitter herbivore damage treatment (two levels: undamaged vs. damaged by *Spodoptera exigua*), fertilization treatment (two levels: unfertilized vs. fertilized), and their interaction on (a) total volatile organic compounds (VOCs, linear mixed model) and (b) VOC composition (PERMANOVA) for potato (*Solanum tuberosum*) plants. In addition, we also show results from linear mixed models testing for effects of the emitter damage treatment (D_E_), fertilization treatments in emitter and receiver (F_E_ and F_R_, respectively) and their two- and three- way interactions (all fixed factors) on (c) the percentage of leaf removed and (d) mass gain of *S. exigua* larvae feeding from a bioassay on receiver plants previously exposed to emitter VOCs. We included individual plant as a random effect (statistics not reported) and height of emitter and receiver plants as a covariate. F /Pseudo-F values for each factor, degrees of freedom (numerator, denominator), and associated *P*-values obtained from the corresponding models are shown. Significant *P*-values are highlighted in bold

	(a) VOC emissions		(b) VOC composition		(c) Percentage of leaf area removed		(d) Larval mass gain	
	F_1,72_	*P*	Pseudo-F_1,72_	*P*	F_1,70_	*P*	F_1,70_	*P*
Emitter herbivore damage (D_E_)	79.1	**< 0.001**	37.79	**< 0.001**	9.38	**0.003**	0.82	0.37
Fertilization in emitters (F_E_)	1.51	0.22	0.53	0.62	0.47	0.49	0.15	0.70
D_E_ × F_E_	0.34	0.56	0.91	0.39	0.05	0.82	0.28	0.59
Fertilization in receivers (F_R_)	-	-	-	-	0.88	0.35	0.01	0.91
D_E_ × F_R_	-	-	-	-	0.22	0.64	0.13	0.71
D_E_ × F_E_ × F_R_	-	-	-	-	0.11	0.74	0.08	0.77
Emitter plant height	9.62	**0.006**	4.66	**0.009**	1.88	0.17	3.58	0.06
Receiver plant height	-	-	-	-	0.01	0.91	0.09	0.77

**Fig. 3:**
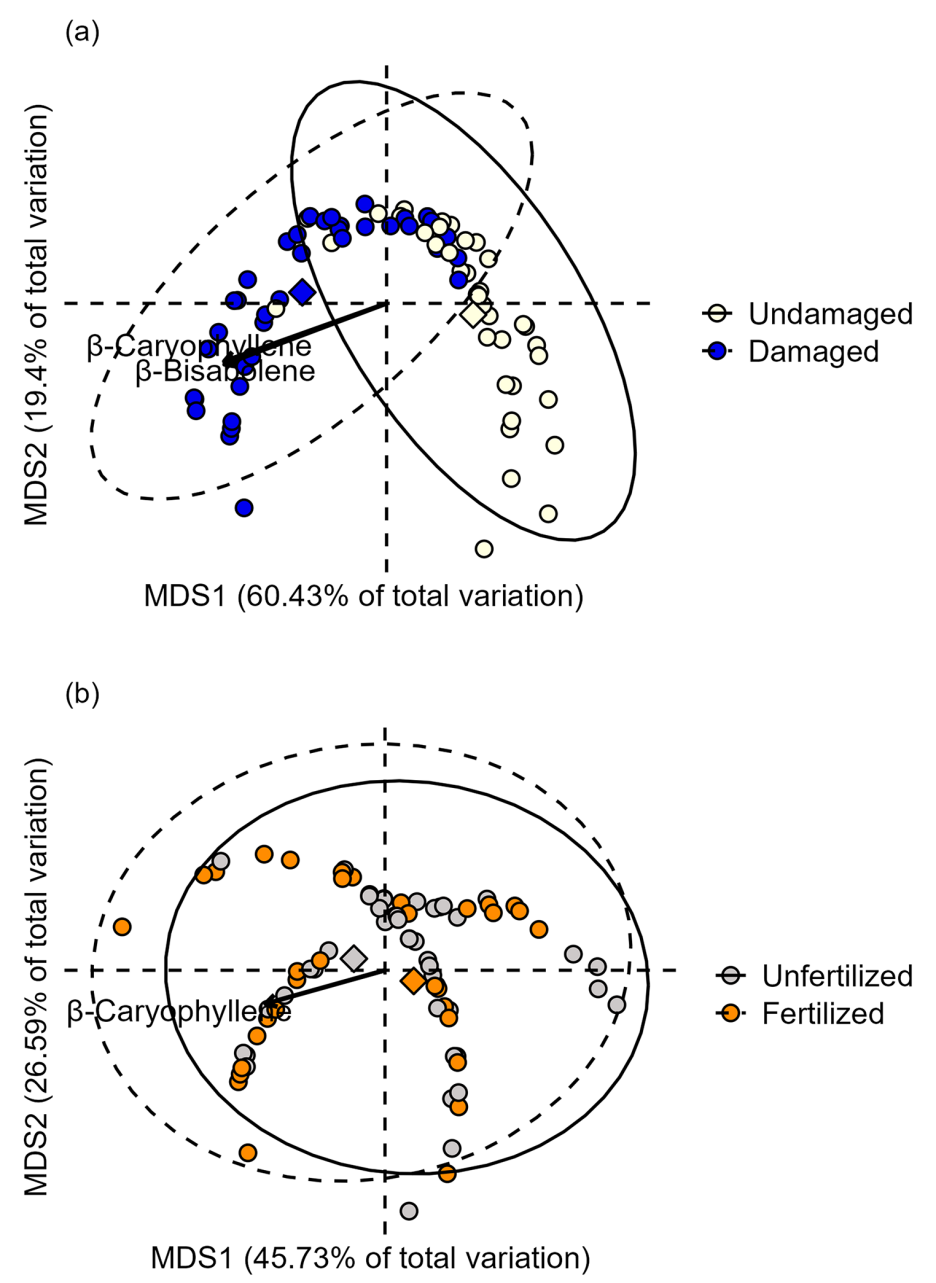
Unconstrained ordination (PCoA) showing the effects of **(a)** emitter herbivore damage treatment (undamaged vs. damaged by *Spodoptera exigua* feeding) and **(b)** fertilization treatment (unfertilized vs. fertilized) on the composition of VOCs released by potato (*Solanum tuberosum*) plants. Biplot arrows represent linear associations with the two most influential volatiles based on their R^2^ values scaled to reflect relative magnitude of effects. Diamonds represent the centroids for each herbivore damage or fertilization treatment and associated 95% ellipses. The first two axes together accounted for 79.46% and 72.4% of total variation in volatile composition due to herbivore damage and fertilization treatments, respectively

Similarly, the PERMANOVA indicated a significant effect of the emitter herbivore damage treatment, but not of nutrient availability or the interaction on VOC composition (Table 1). Emitter damage explained 33% of the variation in VOC composition, with the first two axes of the ordination together accounting for 79.8% of the variation due to this treatment (19.4% and 60.43%, respectively; Fig. 3a). Variation in VOC composition due to emitter damage was mainly associated with the relative amount of β-caryophyllene (R^2^ = 0.85, *P* < 0.001) and β-bisabolene (R^2^ = 0.86, *P* < 0.001). In contrast, fertilization only explained 0.05% of the variation in VOC composition, with the first two axes together accounting for 72.32% of the variation in VOCs due to this factor (26.59% and 45.73% respectively) (Fig. 3b). In addition, the interaction between emitter herbivore damage and fertilization explained a 0.1% of the variation in VOC composition.

### Effects of Herbivore Damage and Soil Nutrients on Signalling and Receiver Resistance

**Fig. 4:**
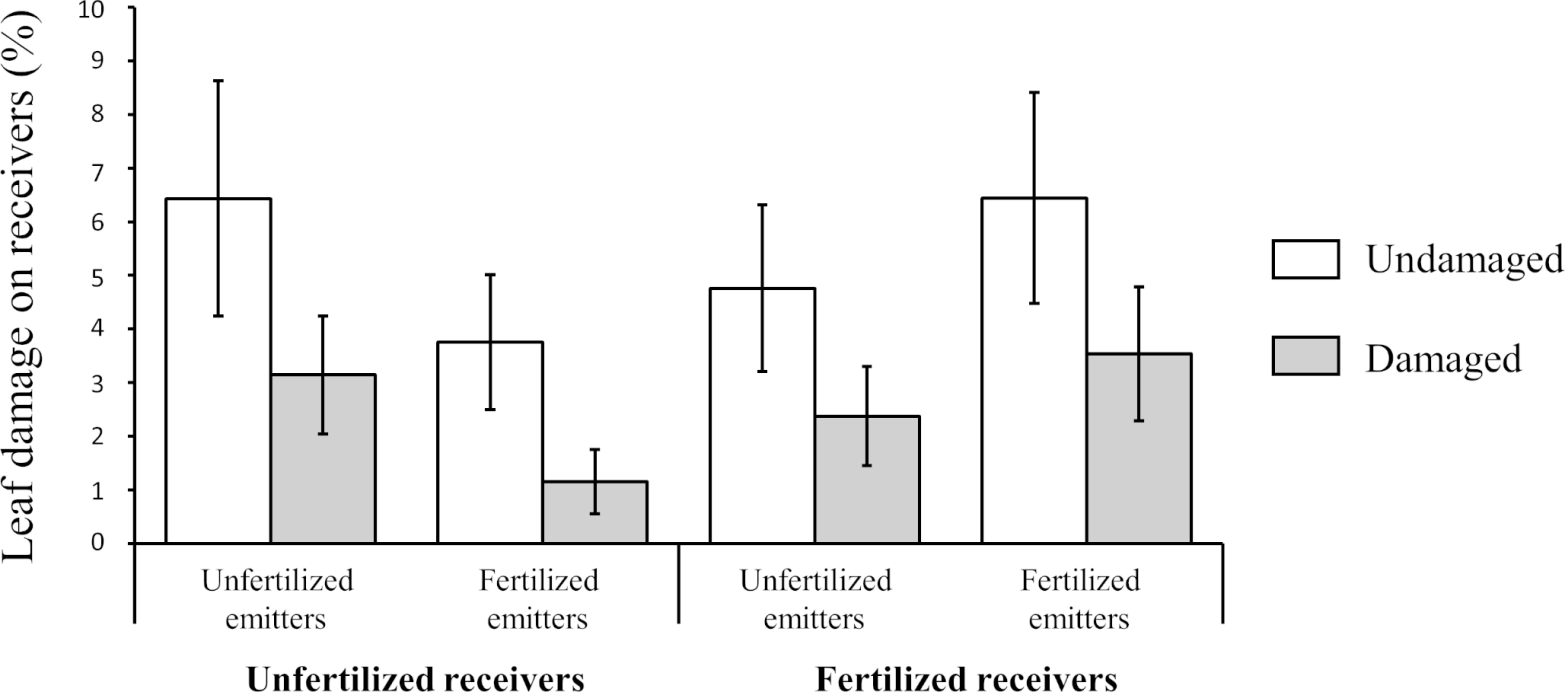
Percentage of leaf removed by *Spodoptera exigua* on receiver potato (*Solanum tuberosum*) plants previously exposed to undamaged (white bars) or herbivore-damaged (grey bars) conspecific emitter plants. Foliar damage on unfertilized and fertilized receivers for each emitter herbivore damage by fertilization combination are shown. Values are model back-transformed least square means ± SE (N = 10)

The emitter damage treatment had a significant effect on the percentage of leaf removed by *S. exigua* but not on larval mass gain on receiver plants (Table 1). In the former case, the mean percentage of leaf removed was 54% lower for receiver plants exposed to VOCs from herbivore-damaged emitters compared to receivers exposed to VOCs from undamaged emitters (mean ± SE: undamaged = 5.24 ± 0.84%; herbivore-damaged = 2.42 ± 0.46% of leaf removed for damaged leaves) (Fig. 4). In contrast, emitter and receiver fertilization did not have a significant effect on either percentage of leaf area removed or larval mass gain on receiver plants (Table 1; Fig. 4). Furthermore, we found no significant two-way or three-way interactions between emitter herbivore damage treatment and emitter/receiver fertilization (Table 1; Fig. 4, Fig. S2).

## Discussion

Leaf herbivory by *S. exigua* drove quantitative (total emissions) and qualitative (compositional) changes in VOCs released by emitter potato plants. Nonetheless, these herbivore-induced changes in VOC emissions were not affected by soil fertilization. Following VOC results, we found that the emitter induction treatment boosted herbivore resistance in receivers (reduced percentage of leaf removed by *S. exigua* larvae), but this signalling effect was not contingent on either emitter or receiver fertilization. Collectively, these findings suggest that VOC-mediated signalling between potato plants in response to *S. exigua* damage is robust to changes in soil nutrients.

Emitter plants subjected to *S. exigua* leaf damage exhibited a 2.5-fold increase in total VOCs released as well as significant VOC compositional changes relative to undamaged emitters, corroborating recent results from another study also using this insect to induce potato seedlings (Vázquez-González et al. 2022). Further analyses by individual compounds showed that damaged emitters released higher concentrations of compounds including the homoterpene nonatriene and sesquiterpenes β- elemene, (*E*)-β-farnesene, and β-bisabolene, all of which were also significantly induced by *S. exigua* herbivory in previous studies with potato (Vázquez-González et al. 2022; Martín-Cacheda et al. 2023). In particular, β-bisabolene, nonatriene, and (*E*)-β-farnesene are known or suspected to be toxic or repellent compounds against phytophagous insects, as well as mediate plant-plant signalling (reviewed by Rosenkranz et al. 2021). For example, nonatriene and (*E*)-β-farnesene were found to be particularly abundant in induced VOC blends released by maize plants and possibly related to priming of induced defences and increased resistance to the oriental armyworm *Mythimna separata*, a specialist (Ramadan et al. 2011). Further work testing the effects of these focal compounds and of VOC blends using different compound ratios or compositions (i.e., mimicking induced blends) are needed to elucidate their role as cues in plant-plant signalling in response to damage by *S. exigua* and other insect pests on potato as well.

We found no evidence for effects of soil fertilization on total VOC emissions or composition. In addition, and more importantly, soil fertilization did not influence emitter damage effects on either total VOCs or VOC composition. The fact that plants grew more under fertilization, but this did not affect VOC induction suggests that either allocation constraints between growth and defence induction were not present or they were not strong enough to influence VOC induction. The relatively few studies that have tested for nutrient availability effects on VOC induction have reported mixed results. For example, Schmelz et al. (2003) reported that nitrogen deficiency increased herbivore-induced VOC emission in maize. Similarly, Chen et al. (2008) found that nitrogen deficiency in cotton plants increases VOC production in response to *S. exigua* herbivory. However, in agreements with our findings, other studies such as that by Lou and Baldwin (2004) reported no effect of fertilization on VOC induction in response to *Manduca sexta* herbivoryin *Nicotiana attenuata*. Terpenes have high turnover and low storage costs (Björkman and Larsson 1991) which could explain why soil fertilization often does not affect (or in some cases positively influences; see Gouinguené and Turlings 2002) VOC induction compared to other costlier compounds. Further work testing for broader range of nutrient fertilization levels (including nutrient limitation or deficiency) combined with explicit assessments of allocation constraints (i.e., growth-defence trade-offs), ideally under field settings, would be desirable to reach stronger generalizations on soil nutrient effects on potato VOC induction. Likewise, studies testing the effects of individual nutrients (e.g., N vs. P) as well as different nutrient mixtures mimicking realistic cultivation scenarios would be highly informative.

Consistent with emitter VOC results, receiver plants exposed to herbivore-damaged emitters were more resistant to herbivory given by a significantly lower percentage of leaf removed by *S. exigua* compared to receivers exposed to undamaged emitters. Similar results have been reported for other crop and non-crop species including maize, cabbage, and Scots pine (Ton et al. 2007; Peng et al. 2011; Karban et al. 2014; Ninkovic et al. 2021; Yu et al. 2022), as well as our recent work with potato and *S. exigua* (Vázquez-González et al. 2022; Martín-Cacheda et al. 2023). Interestingly, in some of these prior studies we found no evidence of signalling effects between potato plants in response to herbivory by the specialist beetle *Leptinotarsa decemlineata* (Abdala-Roberts et al. 2022) or to infection by the generalist pathogenic fungus *Sclerotinia sclerotiorum* (Moreira et al. 2021). The lack of signalling in such cases was possibly due to plant defence-suppression mechanisms by these attackers.IInterestingly, damage by *S. exigua* could induce VOC blends that affect resistance to these other pests (or vice versa) as shown by Marmolejo et al. (2021), who found that VOCs induced by saltmarsh caterpillars suppressed defences in neighbouring zucchini (*Cucurbita pepo*) plants, leading to greater herbivory by beetles. Accordingly, testing the effects of attacker-induced signalling on heterospecific attackers, either through upregulation or downregulation of neighbour induced defences, represents a worthwhile endeavour in future studies.

Counter to predictions, soil fertilization did not affect plant-plant signalling on receiver resistance to herbivory. On the one hand, with respect to emitter-based VOCs variation, this finding would be expected given the lack of effects of fertilization on emitter VOC induction (suggesting no growth-defence induction trade-offs, see above) as this would presumably lead to a concomitant lack of effect of signalling on receivers. On the other hand, from the receiver perspective, this result indicates that increased plant growth (or any other trait change possibly affecting defence allocation) under fertilization did not affect receiver responses to VOCs in terms of induced resistance and its underlying induced defences. This suggests that growth-defence allocation constraints due to fertilization were not present or weak at the most in receivers, which agrees with previous studies reporting no effect of fertilization on direct (Ohnmeiss and Baldwin 1994; Messina et al. 2002; Hahn et al. 2021) or indirect (recruitment of predators; Winter and Rostás 2010; Becker et al. 2015) defences against herbivores. Our study expands on these findings by reporting on fertilization effects from the perspective of VOC-mediated plant-plant signalling. While it is of course plausible that the lack of fertilization effects on emitter VOC induction was enough to preclude any downstream effect of fertilization on receiver-based sources of variation in signalling, effects of receiver fertilization on the “reception” side of signalling cannot be discarded and should be disentangled from effects on VOC emissions in future studies.

Collectively, our results show that signalling between potato plants in response to *S. exigua* damage remains unaltered by increases in soil nutrients, and this appears to be the case in terms of both emitter- and receiver-based variation in responses. Despite this, we believe these findings can inform soil management practices in potato agroecosystems, particularly that aimed at using VOC-mediated signalling for pest control. Two-step evaluations of nutrient increases vs. limitation under varying conditions of water stress or temperature, first under controlled conditions and then in the field, are needed to increase inference and gain knowledge that is more directly applicable for managing crop soils in ways that optimize VOC-mediated signalling and its effects on plant resistance. These studies could be designed (treatments, experimental conditions, etc.) to test for environmental scenarios predicted under climate change.

### Electronic Supplementary Material

Below is the link to the electronic supplementary material.

Supplementary Material 1

## Data Availability

Not applicable.
