# Supplementary material for: Volatile-Mediated Signalling Between Potato Plants in Response to Insect Herbivory is not Contingent on Soil Nutrients
Source: J Chem Ecol. 2023 Jul 18;49(9-10):507–17. doi: 10.1007/s10886-023-01445-y (PMC10725352; doi:10.1007/s10886-023-01445-y)
Supplement: Supplementary file 1 — Supplementary Material 1 [file 10886_2023_1445_MOESM1_ESM.docx]

**Table S1.** Means (± SE) for emission of individual volatile organic compounds (tetralin-equivalent ng h^-1^) identified by GC-MS under two emitter herbivore damage treatments (undamaged vs. damaged by *Spodoptera exigua*) in *Solanum tuberosum* (potato) plants ^1^. RT = Retention times. KRI = Kovats retention index used for identification of compounds without commercial standards (KRI_c_ for calculated values and KRI_e_ for expected values from the NIST database).

| **Compound** | **RT** | **KRI_c_** | **KRI_e_** | **Undamaged** | **Herbivore-damaged** | ***P-*value** |
| --- | --- | --- | --- | --- | --- | --- |
| α-Pinene | 9.67 | 934.37 | 937 ± 3 | 6.29 ± 0.87 | 17.28 ± 2.01 | **<0.001** |
| (-)-β-Pinene | 11.03 | 975.33 | 978 ± 3 | 2.22 ± 0.39 | 6.95 ± 0.88 | **<0.001** |
| β-Myrcene† | 11.51 |  |  | 4.99 ± 0.59 | 8.48 ± 0.69 | **<0.001** |
| p-Cymene | 12.59 | 1023.16 | 1025 ± 2 | 0.26 ± 0.09 | 1.48 ± 0.34 | **<0.001** |
| D-Limonene | 12.78 | 1029.09 | 1030 ± 2 | 2.92 ± 0.89 | 10.07 ± 2.07 | **<0.001** |
| Nonatriene (= (*E*)-4,8-dimethyl-1,3,7-nonatriene) | 15.56 | 1116.62 | 1116 | 0.33 ± 0.16 | 0.68 ± 0.15 | **0.032** |
| Butanoic acid, 3-hexenyl ester, (*E*)- | 17.694 | 1186.04 | 1185 | 0 ± 0 | 1.1 ± 0.21 | **<0.001** |
| Dodecane† | 18.061 |  |  | 3.56 ± 0.26 | 5.21 ± 0.48 | **0.003** |
| Tridecane† | 20.928 |  |  | 1.92 ± 0.19 | 3.7 ± 0.42 | **<0.001** |
| δ-Elemene | 22.011 | 1338.86 | 1338 | 2.08 ± 0.27 | 8.99 ± 1.11 | **<0.001** |
| α-Copaene | 23.079 | 1377.98 | 1376 ± 2 | 1.57 ± 0.23 | 4.28 ± 0.49 | **<0.001** |
| β-Elemene | 23.467 | 1392.19 | 1391 | 13.89 ± 2.33 | 64.52 ± 7.6 | **<0.001** |
| (+)-Sativene | 23.624 | 1397.94 | 1396 | 3.57 ± 0.38 | 8.1 ± 0.82 | **<0.001** |
| α-Gurjunene† | 23.92 |  |  | 12.32 ± 1.51 | 28.88 ± 3.09 | **<0.001** |
| β-Caryophyllene | 24.201 | 1420.56 | 1419 | 133.55 ± 16.3 | 412.4 ± 44.15 | **<0.001** |
| β-Copaene | 24.472 | 1431.25 | 1432 | 1.01 ± 0.16 | 4.26 ± 0.63 | **<0.001** |
| trans-α-Bergamotene | 24.674 | 1439.23 | 1440 | 3.66 ± 0.49 | 12.9 ± 1.59 | **<0.001** |
| cis-β-farnesene | 24.804 | 1444.36 | 1445 | 7.42 ± 0.98 | 22.18 ± 3.15 | **<0.001** |
| trans-Geranylacetone | 25.008 | 1452.40 | 1453 | 0.29 ± 0.07 | 1.17 ± 0.13 | **<0.001** |
| (*E*)-β-Farnesene† | 25.117 |  |  | 24.97 ± 3.12 | 106.72 ± 20.93 | **<0.001** |
| Patchoulene | 25.378 | 1467.01 | 1467 | 8.61 ± 1.01 | 23.22 ± 2.86 | **<0.001** |
| Acoradien | 25.43 | 1469.06 | 1471 | 3.62 ± 0.58 | 13.92 ± 1.57 | **<0.001** |
| Germacrene D | 25.789 | 1483.22 | 1481 | 18.19 ± 2.65 | 87.36 ± 13.65 | **<0.001** |
| Zingiberene | 26.078 | 1494.63 | 1495 | 4.08 ± 0.48 | 14.5 ± 2.16 | **<0.001** |
| Bicyclogermacrene | 26.151 | 1497.51 | 1496 | 7.36 ± 0.94 | 28.33 ± 3.69 | **<0.001** |
| β-Bisabolene | 26.423 | 1508.66 | 1509 | 5.91 ± 0.96 | 25.95 ± 2.75 | **<0.001** |
| γ-Cadinene | 26.596 | 1515.83 | 1513 | 2.52 ± 0.32 | 9.31 ± 1.12 | **<0.001** |
| β-Sesquiphellandrene | 26.794 | 1524.04 | 1524 | 16.37 ± 2.07 | 74.26 ± 11.58 | **<0.001** |
| α-Cadinene | 27.163 | 1539.32 | 1538 | 0.86 ± 0.13 | 3.07 ± 0.37 | **<0.001** |
| Palustrol | 27.909 | 1570.24 | 1568 | 1.53 ± 0.27 | 4.82 ± 0.69 | **<0.001** |
| Unidentified sesquiterpenoid (C_15_H_26_0) | 28.114 |  |  | 35.23 ± 4.47 | 142.99 ± 20.8 | **<0.001** |
| Caryophyllene oxide† | 28.281 |  |  | 2.42 ± 0.39 | 4.72 ± 0.62 | **<0.001** |
| Viridflorol | 28.896 | 1611.86 | 1611 | 8.95 ± 1.06 | 32.25 ± 4.39 | **<0.001** |

^1^We performed *P*-value adjustments using the False Discovery Rate for *P* < 0.05 to avoid inflating Type I error due to multiple testing. Individual VOCs that significantly differed within treatments (*P* < 0.05) are in bold.

^†^Compounds identified with commercial pure standards.


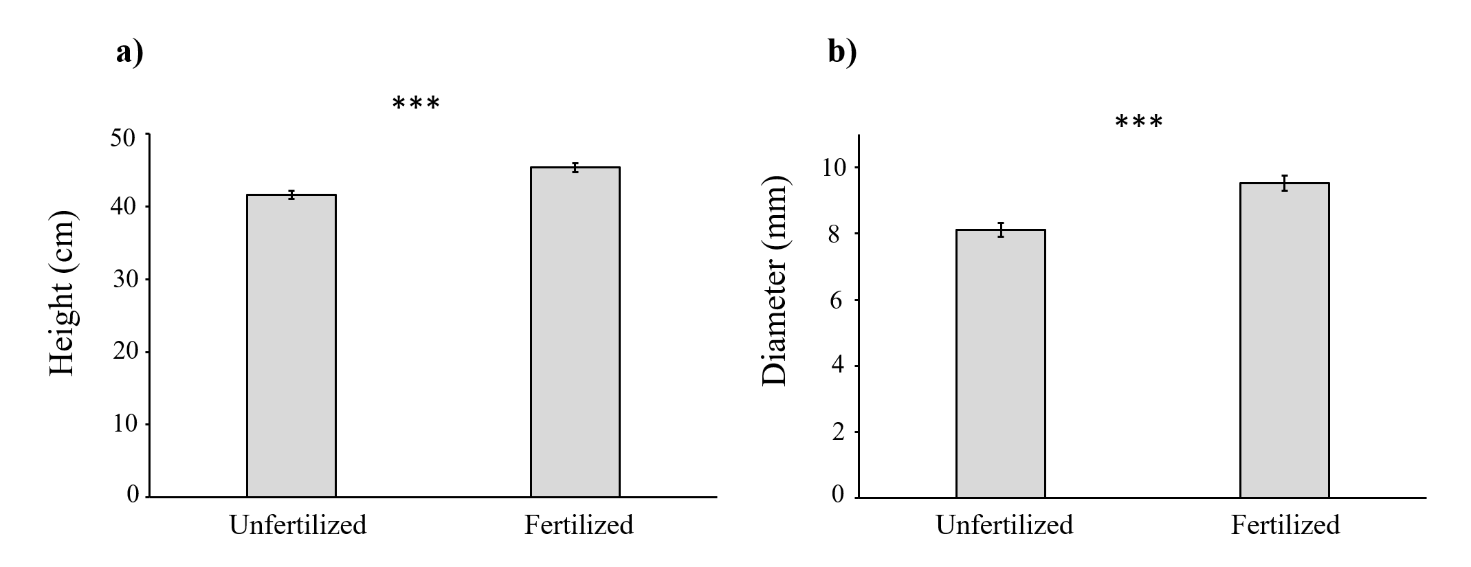


**Figure S1.** Effects of soil fertilization (two levels: unfertilized vs. fertilized) on (a) height and (b) diameter of *Solanum tuberosum* plants. Bars are least square means ± SE (N = 40). Asterisks indicate significant differences between fertilization treatments (****P* < 0.001).


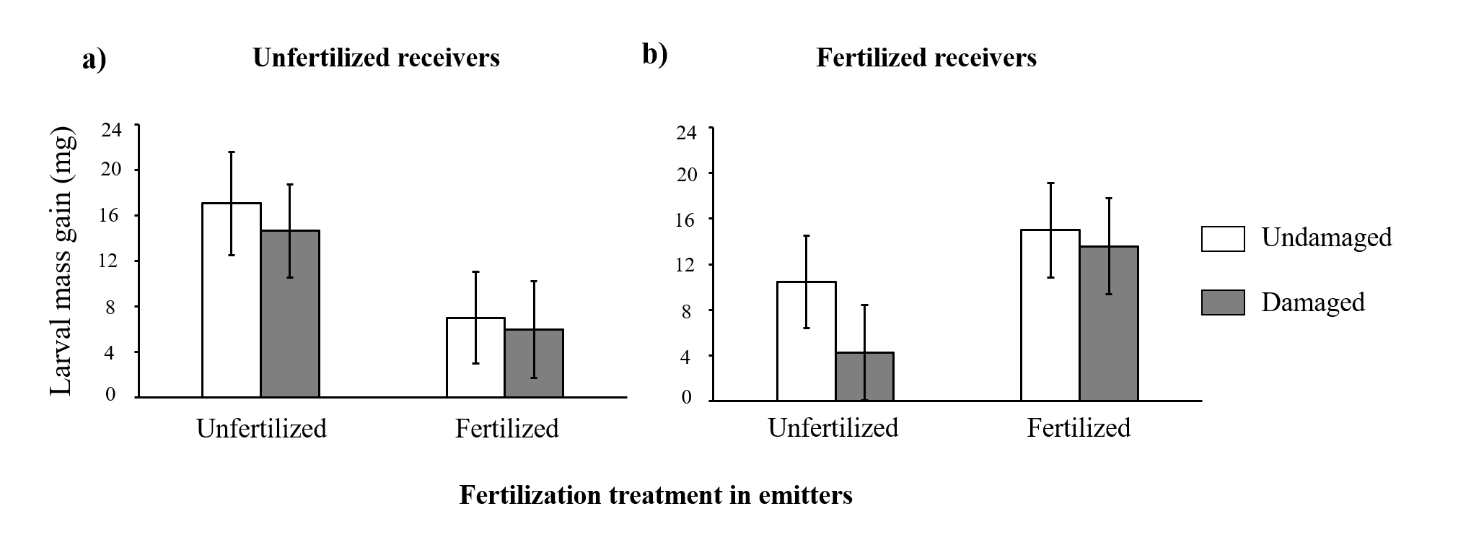


**Figure S2.** Larval mass gain (in mg) on receiver potato (*Solanum tuberosum*) plants previously exposed to undamaged (white bars) and herbivore-damaged (grey bars) conspecific emitter plants. Larval mass gain on (a) unfertilized and (b) fertilized receivers for each emitter induction by fertilization combination. Values are model back-transformed least square means ± SE (N = 10).
